# Toward the Application of High Frequency Electromagnetic Wave Absorption by Carbon Nanostructures

**DOI:** 10.1002/advs.201801057

**Published:** 2019-02-10

**Authors:** Qi Li, Zheng Zhang, Luping Qi, Qingliang Liao, Zhuo Kang, Yue Zhang

**Affiliations:** ^1^ State Key Laboratory for Advanced Metals and Materials School of Materials Science and Engineering University of Science and Technology Beijing Beijing 100083 China; ^2^ Beijing Key Laboratory of Advanced Energy Materials and Technologies University of Science and Technology Beijing Beijing 100083 China

**Keywords:** carbon nanostructures, carbon nanotubes, electromagnetic wave absorption, graphene, high frequency

## Abstract

With the booming development of electronic information technology, the problems caused by electromagnetic (EMs) waves have gradually become serious, and EM wave absorption materials are playing an essential role in daily life. Carbon nanostructures stand out for their unique structures and properties compared with the other absorption materials. Graphene, carbon nanotubes, and other special carbon nanostructures have become especially significant as EM wave absorption materials in the high‐frequency range. Moreover, various nanocomposites based on carbon nanostructures and other lossy materials can be modified as high‐performance absorption materials. Here, the EM wave absorption theories of carbon nanostructures are introduced and recent advances of carbon nanostructures for high‐frequency EM wave absorption are summarized. Meanwhile, the shortcomings, challenges, and prospects of carbon nanostructures for high‐frequency EM wave absorption are presented. Carbon nanostructures are typical EM wave absorption materials being lightweight and having broadband properties. Carbon nanostructures and related nanocomposites represent the developing orientation of high‐performance EM wave absorption materials.

## 1 Introduction

**Figure 1:**
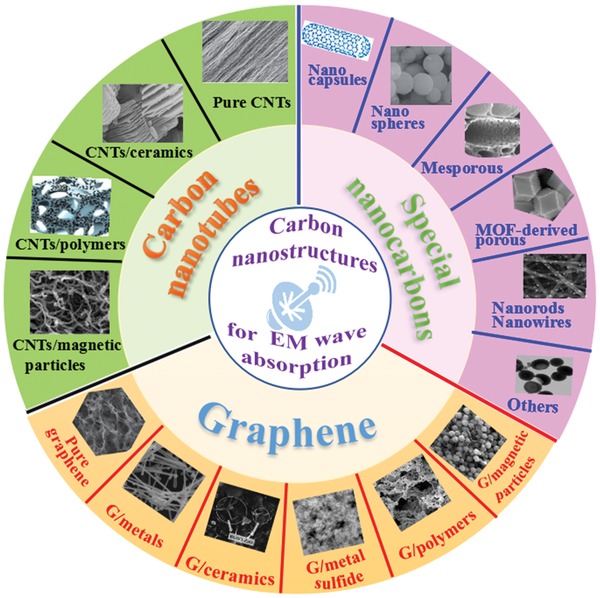
Carbon nanostructures and their composites for high frequency EM wave absorption. Reproduced with permission.^26^ Copyright 2015, Wiley‐VCH. Reproduced with permission.^36^ Copyright 2017, Springer. Reproduced with permission.^37^ Copyright 2015, The Royal Society of Chemistry. Reproduced with permission.^57^ Copyright 2013, American Chemical Society. Reproduced with permission.^88^ Copyright 2014, The Royal Society of Chemistry. Reproduced with permission.^94^ Copyright 2017, The Royal Society of Chemistry. Reproduced with permission.^110^ Copyright 2014, Wiley‐VCH. Reproduced with permission.^124^ Copyright 2017, The Royal Society of Chemistry. Reproduced with permission.^143^ Copyright 2016, The Royal Society of Chemistry. Reproduced with permission.^153^ Copyright 2015, American Chemical Society. Reproduced with permission.^159^ Copyright 2014, The Royal Society of Chemistry. Reproduced with permission.^162^ Copyright 2017, American Chemical Society. Reproduced with permission.^163^ Copyright 2008, American Institute of Physics. Reproduced with permission.^164^ Copyright 2012, The Royal Society of Chemistry.

Rapid development of electronic technology leads to the wide use of electromagnetic (EM) waves in both civil and military fields. The problems of EM interference are very severe due to the broad application of EM waves in the high‐frequency range. The EM interference not only cause damages to telecommunication and electronic devices but also has adverse effects on human health. Therefore, it is important to solve the problems of EM interference by EM wave absorption and shielding. The studies of EM wave absorption materials have attracted much attention all over the world. In recent years, the development of high‐performance EM wave absorption materials being lightweight, and having thinness, broadband, and strong absorption became the most important targets. Until now, some dielectric or magnetic loss materials have played a vital role in high‐frequency EM wave absorption. However, the shortcomings including high density, weak absorption performance, and narrow absorption bandwidth have greatly restricted traditional loss materials' practical applications for EM wave absorption. Nowadays, novel EM wave absorbents are emerging and exhibit outstanding EM wave absorbing properties. Carbon nanostructures have low density, high specific surface area, high permittivity, and excellent electronic conductivity. Therefore, the carbon nanostructures are often designed to meet the demands of high‐performance EM wave absorption material. The carbon nanostructures such as graphene,^1^, ^2^, ^3^ carbon nanotubes (CNTs),^4^, ^5^, ^6^ fullerene,^7^, ^8^ and others^9^, ^10^, ^11^ have a privileged position in the field of high‐frequency EM wave absorption. The carbon nanostructures and related nanocomposites for high‐frequency EM wave absorption are shown in **Figure** **1**.

Usually, the carbon nanostructures for EM wave absorption can be mainly divided into three categories: graphene, CNTs, and other special carbon nanostructures. As the most typical two‐dimensional carbon nanostructures, graphene has sparked extensive interests in EM wave absorption owing to its excellent electron mobility, high thermal conductivity, excellent mechanical properties, and high specific surface area.^1^, ^2^, ^3^ CNTs are the most representative one‐dimensional (1D) carbon nanostructures and possess extremely high mechanical strength, good electrical conductivity, and excellent thermal conductivity. Because of their excellent properties, CNTs have great potential to be used as outstanding EM wave absorption materials.^4^, ^5^, ^6^ Besides, the other special carbon nanostructures could be considered as effective dielectric alternatives as a result of their notable performance of high dielectric loss, low density, and excellent stability.^10^, ^11^ The EM wave absorption of carbon nanostructures are contributed to its dielectric loss. The nonmagnetism of carbon nanostructures limits their EM wave absorption efficiency. Therefore, various nanocomposites based on carbon nanostructures and other lossy materials could be modified as high‐performance EM wave absorption materials. The nanocomposites based on carbon nanostructures can make for better impedance matching, improve the EM wave absorption property, and reduce the density. Furthermore, the designs of nanocomposites based on carbon nanostructures are introduced for high‐performance EM wave absorption materials.

**Figure 2:**
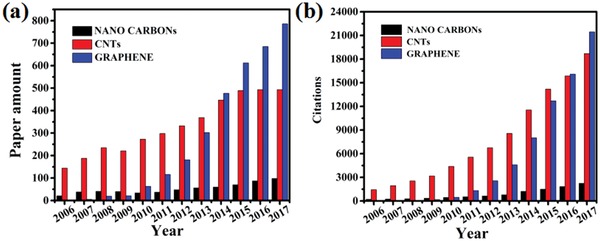
a) The paper amount of carbon nanostructures materials for EM wave absorption 2006–2017, and b) the corresponding citations in 2006–2017.

Carbon nanostructures for EM wave absorption have triggered extensive attention in the past decades. The published papers and the corresponding citations about carbon nanostructures for EM wave absorption have sharply increased year by year. The statistical data of *Web of Science* are shown in **Figure** **2**. The EM wave absorption materials based on carbon nanostructures have become a research hotspot for scientists around the world.

## 2 EM Wave Absorption Theory of Carbon Nanostructures

When the EM wave enters into lossy material, the incident power generated from the EM wave can be divided into three parts: the reflected power, the absorbed power, and the transmitted power.^12^ The reflections of the EM wave consist of surface reflection and multiple reflections. The multiple reflections extend the transmitted routes of the EM wave, and further enhance the absorbing ability of the absorbents.^13^ There are two effective methods to promote the EM wave absorption performance of absorbents. The first method is to increase the transmitted routes of the EM wave in absorbent. The second method is to modulate the EM parameters of absorbents. Relative complex permittivity (ε_r_ = ε′ − jε″) and relative complex permeability (*µ*_r_ = *µ*′ − j*µ*″) determine EM wave absorption performance of absorbents. The terms of ε′ and *µ*′ associate with energy storage. On the contrary, ε'' and *µ*″ stand for the energy dissipation.^14^ The dielectric loss tangent (tan δ_E_ = ε″/ε′) and magnetic loss tangent (tan δ_M_ = *µ*″/*µ*′) indicate the dielectric and magnetic loss capabilities of the EM wave absorbents, respectively. The dielectric loss capabilities mainly are generated from polarization loss, and the polarizations include electronic polarization, dipole polarization, and interfacial polarization. Magnetic losses are caused mainly by magnetic hysteresis, eddy current losses, domain wall resonance, exchange resonance, and natural resonance. The tangentδ can be expressed in the following Equation (1), 13, 14:

(1) $$\tan\delta =\tan\delta_{E}+\tan\delta_{M}=\varepsilon^{\prime \prime }/\varepsilon^{\prime \prime }+\mu^{\prime \prime }/\mu^{\prime }$$

The reflection loss (RL) represents the EM wave absorption ability that can be calculated by the transmission line theory, which is summarized as follows^15^, ^16^:

(2) $$Z_{\mathrm{in}}=Z_{0}{\left(\mu_{r}/\varepsilon_{r}\right)}^{1/2}\tanh\left[j\left(2\pi fd{\left(\mu_{r}\varepsilon_{r}\right)}^{1/2}/c\right)\right]$$

(3) $$\mathrm{RL}=20\log\left|\frac{\left(Z_{\mathrm{in}}-Z_{0}\right)}{\left(Z_{\mathrm{in}}+Z_{0}\right)}\right|$$

where *ε_r_* and *µ_r_* are the relative complex permittivity and permeability, respectively. *c* is the velocity of light, *f* is the frequency of EM wave, *d* is the absorbent's thickness, *Z*_in_ is the input impedance of the absorbent, and *Z*_0_ is the impedance of free space. So, the closer the values of *ε_r_* and *µ_r_*, the less reflection and the more RL would be achieved.

Additionally, the impedance matching is a critical factor in effective EM wave absorption. According to Equation (2), the ideal impedance matching characteristic involves a |*Z*_in_/*Z*_0_| value of 1.0, which corresponds to the complete entrance of the incident EM wave without reflection. The EM wave attenuation is a key parameter for excellent absorption materials. The relationship between attenuation constant (α) and frequency could be determined as the following Equation (4), 17, 18, 23:

(4) $$\alpha =\frac{\sqrt{2}}{c}\pi f\;\times \;\sqrt{\left(\mu^{\prime \prime }\varepsilon^{\prime \prime }-\mu^{\prime }\varepsilon^{\prime }\right)+\sqrt{{\left(\mu^{\prime \prime }\varepsilon^{\prime \prime }-\mu^{\prime }\varepsilon^{\prime }\right)}^{2}+{\left(\mu^{\prime }{\varepsilon^{\prime \prime }}^{}+\mu^{\prime \prime }\varepsilon^{\prime }\right)}^{2}}}$$

The residual defects and the groups of graphene are beneficial for the EM wave absorption. Therefore, reduced graphene oxide (RGO) can be used as high‐performance EM wave absorption materials. The residual defects and groups in RGO not only can make better the impedance matching but also introduce electronic dipole polarization relaxation.^18^, ^19^, ^20^, ^21^ CNTs can be considered as rolled up graphene sheets, which have great potentials to be excellent EM wave absorbents. First, CNTs have excellent tunable electrical conductivity due to individual chirality, which can be adjusted by changing their diameter or layers. The conductive loss is an effective type of loss to attenuate EM wave for CNTs. Secondly, the dielectric loss is another loss for the EM wave absorption of CNTs. The dipolar loss of CNTs resulted from the rotation of electric or magnetic dipoles significantly improve the dielectric loss. The CNTs can act as a template to be deposited some metal or semiconductor nanoparticles, which are very significant for adjusting their impedance matching properties.

The special carbon nanostructures are usually incorporated with other dielectric or magnetic absorbents to design EM wave absorption materials.^9^, ^10^, ^11^ The special carbon nanostructures are a large family with many species, such as carbon nanocapsules, carbon nanospheres, mesoporous carbons, metal organic framework (MOF)‐derived nanoporous carbons, carbon nanorods, carbon nanowires, and bowl‐like or rose‐like carbon nanostructures. The microcosmic appearance and physical nature of special carbon nanostructures are related to the EM wave absorption. First, the multiple internal reflections and scattering of the special carbon nanostructures are beneficial for the enhancement of the EM wave absorption performance.^22^ Second, the plenteous defects originating from the functional groups are the major factors for the high dielectric loss.^13^ Third, the combination of other magnetic and dielectric loss absorbents would generate interfacial polarization.^23^

Carbon nanostructures have exhibited more advantages, such as being lightweight, and having excellent chemical resistance, high conductivity, and permittivity. In order to enhance the EM wave absorption properties of carbon nanostructures, much effort has been devoted to the design of nanocomposites based on carbon nanostructure. A better impedance matching and an excellent EM wave absorption performance can be obtained by the combination of carbon nanostructures with other magnetic or dielectric loss materials.

## 3 Graphene and Its Composites for EM Wave Absorption

Not only is pure graphene the most attractive carbon nanostructure as a novel EM wave absorption material. Its composites have also attracted widespread attention. Owing to their outstanding physical and chemical properties, graphene and its composites have great potential to satisfy the demands of being lightweight, and having thinness, and strong absorption.

### 3.1 Graphene for EM Wave Absorption

Because the magnetism of graphene is very weak, the loss of EM wave is mainly achieved by the way of dielectric loss.^24^, ^25^ Impedance matching characteristic is another important element related to EM wave absorption. Due to the defects and functional groups of RGO, RGO has better impedance matching and stronger EM wave absorption than graphite and high purity graphene.^26^, ^27^, ^28^ The maximum RL for RGO was −6.9 dB at 7 GHz, which was stronger than that of graphene oxide and high‐purity graphene.^24^ Cao's work demonstrated that ultrathin graphene exhibited high‐performance EM wave absorption at elevated temperatures. The maximum RL of graphene composites was −42 dB. In addition, the effective absorption bandwidth covered almost the whole X‐band (8–12 GHz).^25^

**Figure 3:**
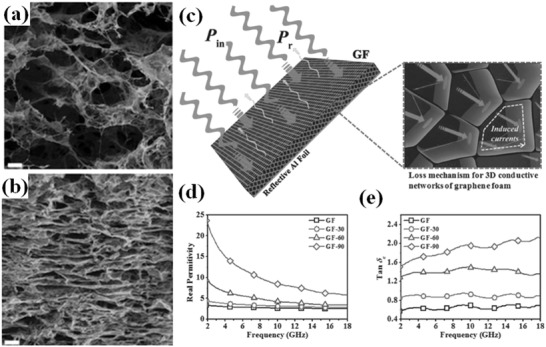
a,b) The cross‐sectional SEM images of GF and GF‐90, the scale bar is 20 µm. c) Schematic representation of the EM absorption mechanism for the GF. d) Real parts of the complex permittivity of the GFs in the range 2–18 GHz. e) Dielectric loss tangent of the GFs in the range 2–18 GHz. Reproduced with permission.^26^ Copyright 2015, Wiley‐VCH.

Lately, significant progress in the synthesis of 3D interconnected graphene networks have been made.^27^, ^28^, ^29^, ^30^ 3D graphene networks still maintain the excellent properties of graphene sheets, which could guarantee promising applications for EM wave absorption.^31^ Furthermore, the numerous homogeneously dispersed internal pores not only led to lower bulk density but also guaranteed lower permittivity, which provided better impedance matching. Zhang et al. synthesized graphene foams (GFs) with different compressive strain. The cross‐sectional scanning electron microscopy (SEM) images and dielectric loss of the compressive GFs are shown in **Figure** **3**. The qualified bandwidth of 60.5 GHz is obtained from compressive GFs, covering 93.8% of the measured frequency bands.^26^ Thermally reduced graphene networks (TRGN) were synthesized by thermal reduction of graphene oxide/poly(vinyl alcohol) networks.^27^ The TRGN/wax composites reached the maximum RL of −43.5 dB at 12.19 GHz, and the bandwidth of RL below −10 dB was 7.47 GHz. The conductive interconnections of 3D graphene lead to lower loading and effective EM wave absorption. 3D honeycomb‐like structure of RGO had excellent EM wave absorption performance in the S‐band (2–4 GHz).^28^ The effective absorption bandwidth covered 2.3–4.1 GHz.

**Figure 4:**
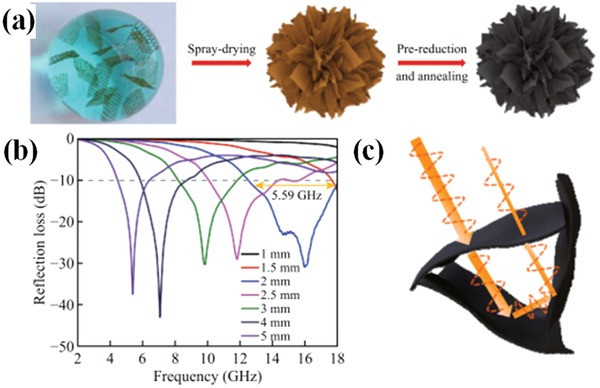
a) Schematic illustration of the formation of Gmfs. b) RL of 10 wt% Gmfs/paraffin composite with various thicknesses at 2–18 GH. c) Schematic illustration of EM absorption mechanism of Gmfs. Reproduced with permission.^29^ Copyright 2018, Springer.

Other 3D structures of graphene have been fabricated by special methods. Chen et al. prepared graphene microflowers with highly porous structure.^29^ The excellent EM wave absorption performance of graphene microflowers are shown in **Figure** **4**. The effective absorption bandwidth of RL below −10 dB is 5.59 GHz and the maximum RL is −42.8 dB.

### 3.2 Binary Graphene Composites for EM Wave Absorption

In order to get better EM wave absorption properties, binary graphene composites are prepared by combining with other loss materials. The composites for EM wave absorption mainly contain conductive metals and alloys, ceramics, transition metal sulfide, magnetic nanoparticles, and so on.

#### 3.2.1 Graphene/Conductive Metal Composites for EM Wave Absorption

Copper and silver metals are the traditional and widespread conductive materials, and they have excellent electrical conductivity and thermal conductivity. EM wave can be absorbed and shielded by the copper and silver films.^32^, ^33^ The maximum RL of RGO/Cu composites was up to −50.4 dB at 3.8 GHz.^32^ Besides, RGO/Ag composites were applied as filler in polymethyl methacrylate (PMMA) matrix. The RGO/Ag composites exhibited an excellent EM shielding effectiveness of 26.8 dB in the X‐band. The primary effect of EM shielding resulted from the shielding effectiveness absorption (SE_A_).^33^ The conductive metal nanoparticles were so small that they gave rise to an increase in the dipoles. In addition, the RGO was decorated by conductive metal nanoparticles and heterogeneous interfaces formed in the fabricated nanocomposites. The enhanced interfacial polarizations contributed to the dielectric loss.

#### 3.2.2 Graphene/Ceramics Composites for EM Wave Absorption

In the different EM wave absorption materials, some ceramics including ZnO,^22^, ^34^, ^35^ MnO_2_,^39^ Mn_3_O_4_,^40^ V_2_O_5_,^41^ SiC,^42^, ^43^ SiO_2_,^44^ and MgO^45^ have outstanding high corrosion resistance, electrical insulation, and superior thermal stability. The ceramics have extensive applications in extremely harsh conditions and exhibit excellent EM wave absorption performance. Numerous graphene/ceramics composites for EM wave absorption were prepared by different methods.

**Figure 5:**
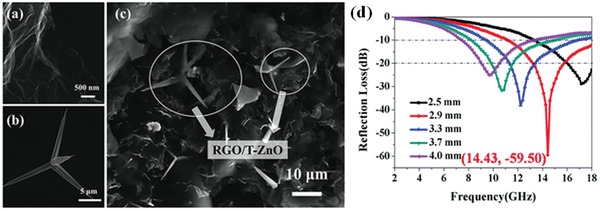
a,b) The SEM images of RGO and T‐ZnO. c) The fractured cross‐section of RGO/T‐ZnO composites with 5 wt% RGO and 10 wt% T‐ZnO whiskers. d) The RL curve of RGO/T‐ZnO composite with 5 wt% RGO and 10 wt% T‐ZnO at 2–18 GHz. Reproduced with permission.^37^ Copyright 2015, The Royal Society of Chemistry.

ZnO, as a wide bandgap semiconductor has been extensively investigated in the field of EM wave absorption. Some studies about EM wave absorption of RGO/ZnO nanocomposites have been reported. Zhang et al. fabricated the RGO/tetrapod‐like ZnO (T‐ZnO) composites.^37^ The nanostructures and the EM absorption properties of composites are shown in **Figure** **5**. The maximum RL of −59.50 dB is achieved at 14.43 GHz.

**Figure 6:**
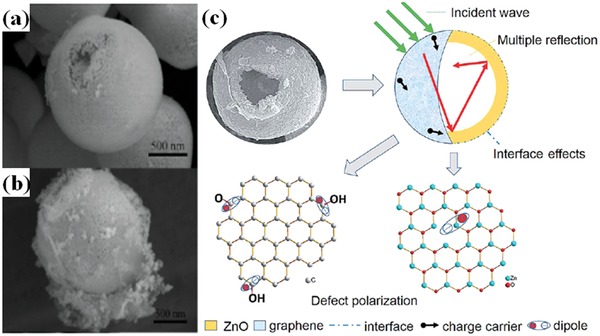
a) SEM image of the as‐prepared ZnO hollow spheres. b) SEM image of graphene/ZnO hollow spheres. c) A schematic approach to enhance the EM wave absorption of graphene wrapped ZnO spheres. Reproduced with permission.^38^ Copyright 2014, The Royal Society of Chemistry.

Other ZnO structures have been investigated, such as ZnO hollow spheres, ZnO nanocrystals, and so on.^34^, ^38^ Graphene‐wrapped ZnO hollow spheres and the EM wave absorption mechanisms are shown in **Figure** **6**.^38^ The graphene‐wrapped ZnO composites exhibit a maximum absorption of −45.05 dB at 9.7 GHz. The combination of graphene nanosheets with different ZnO structures is an effective way to design lightweight EM wave absorbents.

**Figure 7:**
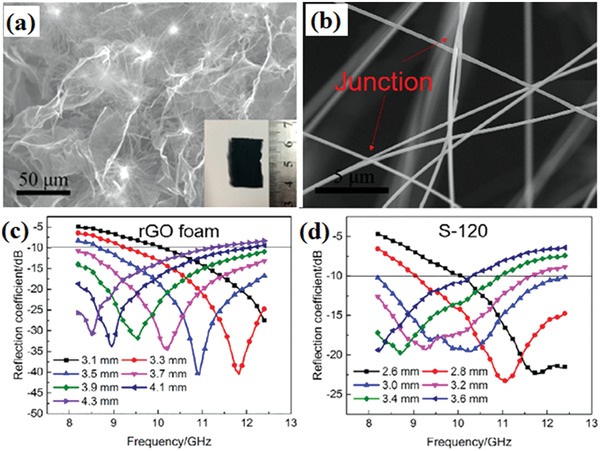
a) SEM image of RGO/SiC NW foam. b) High‐resolution SEM image of typical junction of SiC NWs. c,d) RC values calculated for rGO foam and rGO/SiC NWs foams at different thickness. Reproduced with permission.^42^ Copyright 2017, American Chemical Society.

MnO_2_ offered another pathway for the development of advanced EM wave absorption materials due to its low cost and environment‐friendly characteristics. The maximum EM shielding effectiveness of the graphene nanoribbons decorated with MnO_2_ was 57 dB.^39^ In addition, the most shielding effectiveness came from EM wave absorption. The EM wave absorption performance of Mn_3_O_4_ has been studied.^40^ The RGO/Mn_3_O_4_ composites achieved the maximum RL of −29 dB in 2–18 GHz. V_2_O_5_ is another analogous transition metal oxide with attractive electronic, electrochemical, and electrocatalytic properties. The effective bandwidth of the RL below −10 dB for 3D‐RGO/V_2_O_5_ was 6.63 GHz with the thickness of 2.5 mm.^41^ SiC is a good candidate for EM wave absorption material due to its high strength and excellent thermal stability. Han et al. prepared 3D RGO/SiC composites consisting of RGO and SiC nanowires (NWs).^42^ The RGO/SiC composites and their EM wave absorption performance are shown in **Figure** **7**. The RGO/SiC foams achieve the effective absorption in the whole X‐band. SiC NWs with abundant stacking faults, bridged junctions, and twinning interfaces play an essential role in the enhancement of EM wave absorption performance. In other works, RGO incorporated SiOC composites were fabricated by the in situ grown method. The composites demonstrated strong EM wave absorbing peak of −69.3 dB. Under the high‐temperature environment, the effective absorption bandwidth of the composites with 2 wt% RGO was 3.9 GHz in the X‐band.^43^ The high‐performance EM wave absorption of graphene/ceramics composites are mainly attributed to the excellent impedance matching as well as the dielectric loss.

#### 3.2.3 Graphene/Metal Sulfide Composites for EM Wave Absorption

**Figure 8:**
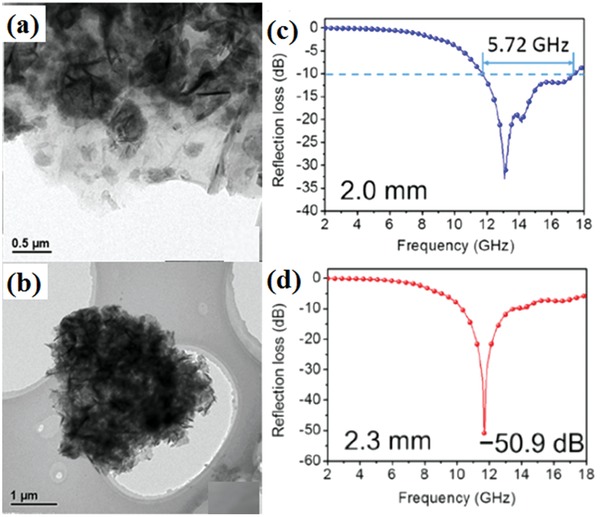
a) TEM image of RGO/MoS_2_. b) TEM image of an agglomerate of MoS_2_ nanosheets. c,d) RL curves of MoS_2_/RGO composites at different thicknesses. Reproduced with permission.^48^ Copyright 2015, American Chemical Society.

Recently, transition metal oxides are widely applied in EM wave absorption. Transition metal sulfide, including CuS,^23^ MoS_2_,^36^, ^46^ and CoS_2_,^47^ also have been considered as novel EM wave absorption materials. Theoretically, metal sulfide possesses better conductive ability than its oxide counterparts due to its relatively smaller bandgap value. Metal sulfide also has stronger electron transmission ability. Therefore, it is rarely used as the loading material with highly conductive graphene. CoS_2_ have attracted particular interests for their unique magnetic and electrical abilities. The maximum RL of RGO/CoS_2_ nanohybrids reached −56.9 dB at 10.9 GHz. Meanwhile, the RL below −10 dB was obtained in the frequency range of 9.1–13.2 GHz.^47^ MoS_2_ was another transition metal dichalcogenides with layered structure. The transmission electron microscope (TEM) images and EM wave absorption performance of RGO/MoS_2_ composites are shown in **Figure** **8**. RGO/MoS_2_ composites exhibit excellent EM wave absorption performance with an effective bandwidth of 5.72 GHz and the maximum RL of −50.9 dB.^48^ Taking the low cost and high stability into account, the metal sulfide are deserved to further investigated.

#### 3.2.4 Graphene/Conductive Polymer Composites for EM Wave Absorption

Graphene/conductive polymer composites possess numerous advantages including being lightweight, and having flexibility, and tunable conductivities. The graphene in graphene/polymer composites are connected with each other to construct a conductive network. Thus, the orientational movement of carriers results in the conductive loss in the changing EM field. Moreover, the dipole formed at the interfaces of graphene/polymers would cause the lattice vibration. The EM wave would be attenuated in the form of heat energy. Furthermore, scattering and multiple reflections could also be obtained owing to the difference of dielectric constant at the interfaces. Therefore, conductive loss, dipole polarization, interfacial, and multiple scattering are the main reasons for EM wave absorption. Recently, polyaniline(PANI),^49^, ^50^ polypyrrole(PPy),^51^, ^56^ nitrile butadiene rubber(NBR),^52^ poly‐(ethylene oxide)(PEO),^53^ poly(3, 4‐ethylene dioxythiophene)(PEDOT),^54^ polyvinylidene fluoride(PVDF)^55^ and polyetherimide(PEI)^57^ have drawn extensive attentions.

**Figure 9:**
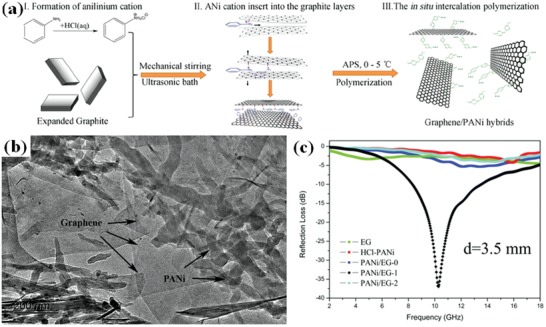
a) Schematic for the intercalation polymerization of ANi^+^ into EG to synthesize graphene/PANI hybrids. b) TEM image of the composites obtained by the intercalation polymerization of ANi^+^ into 1 wt% EG (denoted as PANi/EG‐1). c) Calculated RL of the samples with a thickness of 3.5 mm in the frequency range of 2–18 GH. Reproduced with permission.^50^ Copyright 2014, Wiley‐VCH.

Chen et al. prepared expanded graphene/PANI hybrids.^50^ The prepared graphene/PANI hybrids and EM wave absorption performance are shown in **Figure** **9**. The maximum RL of graphene/PANI hybrids is −36.9 dB, and the effective absorption bandwidth corresponding to the RL below −10 dB is up to 5.3 GHz. The effective absorption bandwidth of RGO aerogel/sponge like PPy composites was 6.76 GHz, and the maximum RL reached −54.4 dB at 12.76 GHz.^51^ The enhanced EM wave absorption properties of graphene/conductive polymers are resulted from the unique structure, the excellent impedance matching and dielectric loss.

**Figure 10:**
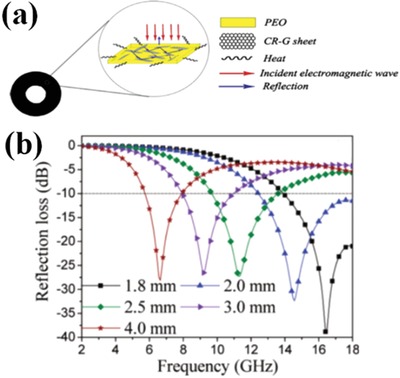
a) A schematic representation for the possible dissipation route of electromagnetic wave in the CR‐G/PEO composites. b) RL curves for the 2.6 vol% CR‐G/PEO composites. Reproduced with permission.^53^ Copyright 2011, American Chemical Society.

NBR have good qualities including corrosion prevention and the properties of lightweight. RGO/NBR composites exhibited high values of RL of −57 dB at 9.6 GHz.^52^ Reduced graphene/PEO composites were prepared by chemical method, and their EM wave absorption performances are shown in **Figure** **10**.^53^ The maximum RL of reduced graphene/PEO composites is −32.4 dB. The EM wave absorption performance of RGO/PEO composites is attributed to electrical conduction loss and dielectric loss. The residual defects and groups of graphene interface are beneficial for the deposition of PEDOT nanofibers.^54^ As for the PEDOT/graphene composites, the maximum value of RL was −48.1 dB.

The combination of graphene and PVDF has been used for novel EM wave absorption materials. The maximum RL of the RGO/PVDF membrane reached −25.6 dB at 10.8 GHz, and the effective absorption band below −10 dB was from 8.48 to 12.80 GHz.^55^ Graphene aerogels were constructed by using the cigarette filters as templates.^56^ The graphene aerogels showed a maximum RL of −30.53 dB at 14.6 GHz, and the absorption bandwidth of RL less than −10 dB was 4.1 GHz. Furthermore, the maximum RL of graphene aerogels reached −45.12 dB by PPy coating.

In conclusion, the enhanced EM wave absorption properties of graphene/conductive polymers composites could be attributed to the covalent anchoring and the synergistic effects. The covalent bond connection changed the electron cloud density of graphene and improved the EM parameters of graphene. Moreover, some conductive polymers with π‐conjugated main chains have a delocalized electronic structure and distinctive electrical properties. The individual structures and properties of conductive polymers provide great opportunities for the design of high‐performance EM wave absorption materials.

#### 3.2.5 Graphene/Magnetic Nanoparticles Composites for EM Wave Absorption

The EM wave absorption mechanism of graphene is dielectric loss. In addition, the EM parameters of graphene are out of balance and bad for impedance matching. Therefore, combination of graphene and magnetic loss components is an effective way to modulate the interfacial polarization and impedance matching. The traditional magnetic metals and oxides such as Fe,^58^, ^67^ Co,^68^, ^69^ Ni,^70^ γ‐Fe_2_O_3_,^59^, ^72^, ^73^ Fe_3_O_4_,^60^, ^76^, ^77^, ^78^, ^79^ NiO,^61^ CoFe_2_O_4_,^62^, ^63^ and NiFe_2_O_4_,^64^ Co_3_O_4_,^80^ possessed large saturation magnetization and high Snoek's limit. The modification of magnetic nanoparticles on graphene surface have been widely investigated in high‐frequency EM wave absorption. The permittivity and permeability of the graphene/magnetic nanoparticles composites are compatible in the gigahertz frequency.

**Figure 11:**
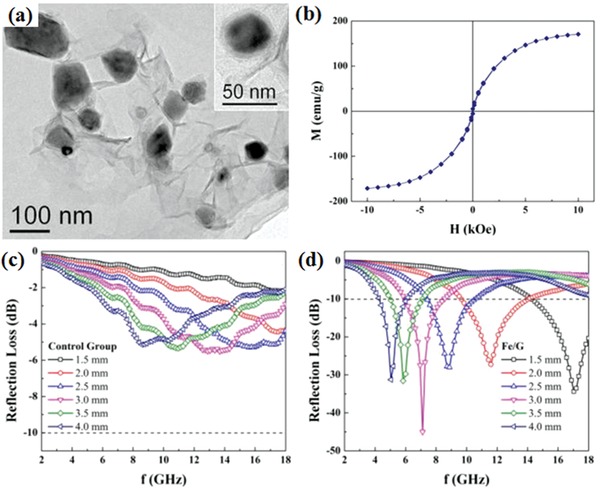
a) TEM image of graphene/Fe, the inset is an enlarged image of individual Fe nanoparticle. b) The magnetic hysteresis loop for graphene/ Fe at room temperature. c,d) Calculated RL of different thickness for control group and graphene/Fe. Reproduced with permission.^65^ Copyright 2013, Nature Publishing Group.

The microstructures and EM wave absorption performance of prepared graphene/Fe composites are shown in **Figure** **11**.^65^ The maximum RL is −45 dB at 7.1 GHz. Meanwhile, the effective absorption bandwidth of the graphene/Fe composites is up to 4.4 GHz. The graphene/magnetic metal composites have some disadvantages, such as easy to corrode and large dielectric constant. To overcome the disadvantages, many investigations on graphene/magnetic metal oxide composites were carried out. The 3D graphene/γ‐Fe_2_O_3_ nanosheet array hetero‐nanostructures were fabricated by a seed‐assisted method.^71^

**Figure 12:**
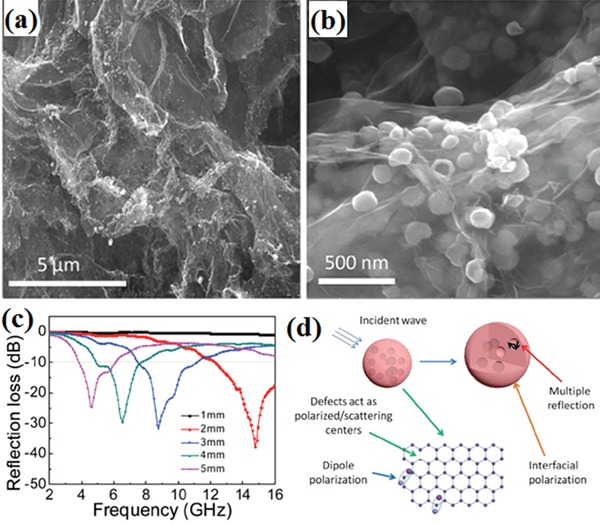
a) SEM image of inter‐connected 3D network microstructure RGO/Fe_2_O_3_ composites. b) An enlarged SEM image of Fe_2_O_3_ nanoparticles attached on the surface or encapsulated inside the graphene sheets. c) The RL of RGO/Fe_2_O_3_ composites. d) Schematic diagram for possible EM absorption mechanism of rGO/Fe_2_O_3_ composites. Reproduced with permission.^72^ Copyright 2015, The Royal Society of Chemistry.

The microstructures and EM wave absorption performance of RGO/γ‐Fe_2_O_3_ composites are shown in **Figure** **12**.^72^ The maximum RL of porous RGO/γ‐Fe_2_O_3_ composites is −38 dB at 14.78 GHz. The effective absorption bandwidth is up to 5.8 GHz. The excellent EM wave absorption performance of RGO/γ‐Fe_2_O_3_ is related to special structures and the synergetic effects. The maximum RL of prepared RGO/Fe_3_O_4_ composites was −49.05 dB at 14.16 GHz.^74^ The effective absorption bandwidth was about 4.60 GHz. The maximum RL of bowl‐like hollow RGO/Fe_3_O_4_ composites was −24 dB and the effective EM wave absorption bandwidth was up to 4.9 GHz.^75^

**Figure 13:**
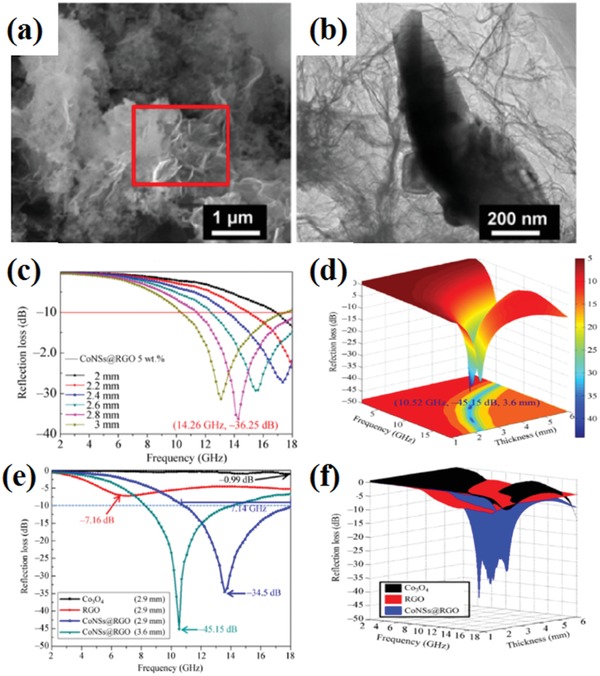
a) Typical SEM images of CoNSs@RGO. b) TEM images of CoNSs@RGO; c) The RL of CoNSs@RGO 5wt%. d) 3D plots of the RL and EM wave frequency of sample CoNSs@RGO 5wt%. e) The RL of Co_3_O_4_ 5 wt%, CoNSs @ RGO 5 wt% and RGO 2.5 wt%. f) 3D plots of the RL and EM wave frequency of sample Co_3_O_4_ 5 wt%, CoNSs@RGO5 wt%, and RGO 2.5 wt%. Reproduced with permission.^81^ Copyright 2017, Springer.

Metal cobalt as well as cobalt oxides also could be designed as EM wave absorption materials. The maximum RL value of RGO/Co_3_O_4_ composites was −43.7 dB. The EM wave absorption bandwidth with the RL below −10 dB was up to 4.6 GHz.^80^ The Co_3_O_4_ nanosheets(CoNSs)@RGO were prepared by three‐step chemical method.^81^ The CoNSs@RGO composites and their EM wave absorption performance are shown in **Figure** **13**. The maximum RL CoNSs@RGO composites is −45.15 dB at 10.52 GHz. Furthermore, the effective EM wave absorption bandwidth of the composites is 7.14 GHz.

**Figure 14:**
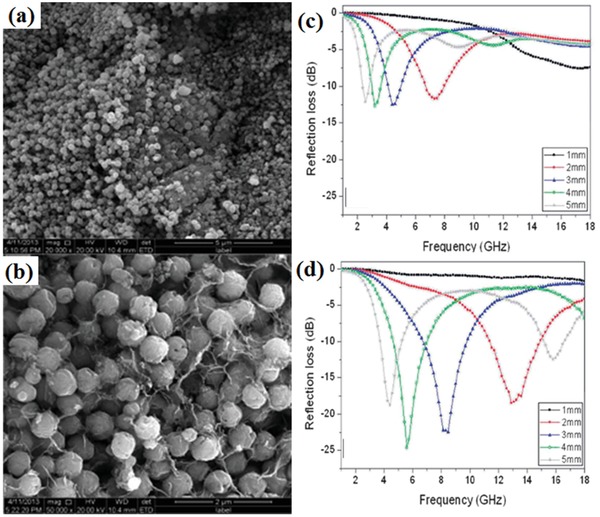
a) SEM images of CoFe_2_O_4_ hollow spheres. b) Graphene/ CoFe_2_O_4_ hollow sphere composites. c) The calculated RL of the CoFe_2_O_4_ hollow spheres. d) The calculated RL of graphene/CoFe_2_O_4_ hollow sphere composites. Reproduced with permission.^88^ Copyright 2014, The Royal Society of Chemistry.

The EM wave absorption performance of RGO/Ni composites were mainly resulted from the synergistic effect of dielectric loss and magnetic loss.^66^ The maximum RL of RGO/Ni composites was −42 dB at 17.6 GHz. Wang et al. synthesized novel porous flower‐like NiO@graphene by a facile hydrothermal reaction and an annealing process.^82^ The maximum RL of porous flower‐like NiO@graphene was −59.6 dB and the effective EM wave absorption bandwidth covered 4.2 GHz. Other magnetic particles including hexagonal BaFe_12_O_19_ 84, 85 and carbonyl iron^83^, ^86^ were widely designed as the EM wave absorption materials. Zhu et al. investigated the EM wave absorption performance of the RGO/spherical carbonyl iron composites (RGO/SCI).^83^ The maximum RL and the effective EM wave absorption bandwidth of RGO/SCI composites reached −52.46 dB and 4.19 GHz, respectively. The maximum RL of graphene/Ba_0.8_La_0.2_Fe_12_O_19_ composites was −40.26 dB and the related effective EM wave absorption bandwidth was 3.87 GHz.^84^ Magnetic CoFe_2_O_4_ nanoparticles had large magnetocrystalline anisotropy and high saturation magnetization. The functionalized RGO/cobalt ferrite composites were prepared by a three‐step chemical method.^87^ The effective EM wave absorption bandwidth of the composites was 7.17 GHz. Fu et al. synthesized graphene/hollow sphere CoFe_2_O_4_ nanocomposites by a simple vapor diffusion and calcination method.^88^ The formation schematic diagram and EM wave absorption performance of the graphene/hollow sphere CoFe_2_O_4_ composites are shown in **Figure** **14**. The maximum RL of the prepared composites is −18.5 dB and the effective absorption bandwidth is 3.7 GHz. The maximum RL of RGO/NiFe_2_O_4_ composites was −42 dB and the effective EM wave absorption bandwidth was 5.3 GHz.^89^ The EM wave absorption performance of RGO/CoFe_2_O_4_ were attributed to the dielectric polarization and the hysteresis loss. Moreover, the composites had much better impedance matching.

### 3.3 Ternary Graphene Composites for EM Wave Absorption

The further developments of EM wave absorbents have focused on the performance of multicomponent graphene composites. Different multicomponent graphene composites, such as Ba_0.9_La_0.1_Fe_11.9_Ni_0.1_O_19_/RGO/polyaniline(BF/RGO/PANI),^85^ RGO/Cu_2_O/Cu,^91^ Ag@Fe_3_O_4_/RGO,^92^ Cu@Ni/GO,^93^, ^94^ FeNi alloy/carbon porous microspheres/RGO (FeNi/CS/RGO),^95^ FeCo@RGO@PPy,^96^ FeNi_3_@SiO_2_@RGO/PANI,^97^ graphene/PPy/CoFe_2_O_4_,^99^ graphene/PANI/CoFe_2_O_4_ 100 were developed. These absorbents have similar EM wave absorption mechanisms, and the coupling of different absorbents enhance the attenuation intensity.^90^

**Figure 15:**
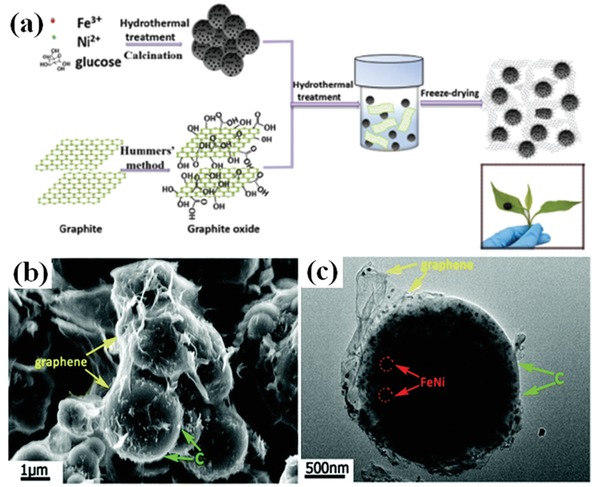
a) Schematic illustration of the formation of the FeNi/CS/rGO composite. b) SEM image of FeNi/CS/rGO. c) TEM image of FeNi/CS/rGO. Reproduced with permission.^95^ Copyright 2017, The Royal Society of Chemistry.

RGO/Cu_2_O/Cu composites were synthesized by a mild wet‐chemical reactions.^91^ The maximum RL of the composites was −51.8 dB and the effective EM wave absorption bandwidth was 4.1 GHz. Ag@Fe_3_O_4_/RGO composites had the maximum RL of −40.05 dB and the effective EM wave absorption bandwidth was 3.1 GHz.^92^ The EM wave absorption of Cu@Ni/RGO was resulted from the synergistic effects of the dielectric loss and magnetic loss.^94^ Wang et al. synthesized Cu@Ni NWs/RGO composites.^93^ Compared to Cu@Ni NWs or pure rGO, the Cu@Ni NWs/RGO composites had the highest RL value of −42.8 dB. The FeNi/CS/RGO composites were fabricated by the chemical method.^95^ The microstructures and EM wave absorption properties of the fabricated composites are shown in **Figure** **15**. The maximum RL value of the FeNi/CS/RGO composites is −45.2 dB and the effective EM wave absorption bandwidth is 5.0 GHz.

The novel ternary graphene/conductive polymers including PANI^49^, ^50^ and PPy^51^, ^56^ have been fabricated by many methods. Wang et al. synthesized ternary sandwich structure of FeCo@RGO@PPy nanocomposites.^96^ The maximum RL of FeCo@RGO@PPy nanocomposites was −40.7 dB and the effective EM wave absorption bandwidth was 5.7 GHz. Ding et al. synthesized FeNi_3_@SiO_2_@RGO/PANI multicomponent composites and the maximum RL of the multicomponent composites reached −40.18 dB.^97^ The maximum RL of the PPy/RGO/Co_3_O_4_ nanocomposites was up to −33.5 dB at 15.8 GHz.^98^ In addition, graphene/PPy/CoFe_2_O_4_ 99 and graphene/PANI/CoFe_2_O_4_ 100 composites all had excellent EM wave absorption performances. The maximum RL of graphene/PPy/CoFe_2_O_4_ composites was −50.8 dB. Compared to graphene/PANI/CoFe_2_O_4_ composites, the PPy incorporated RGO/CoFe_2_O_4_ composites had more excellent EM wave absorption performance. Ba_0.9_La_0.1_Fe_11.9_Ni_0.1_O_19_/RGO/PANI composites were prepared by in situ polymerization.^85^ The maximum RL of composites reached −49.1 dB and the effective EM wave absorption band was from 12.4 to 16.72 GHz.

**Figure 16:**
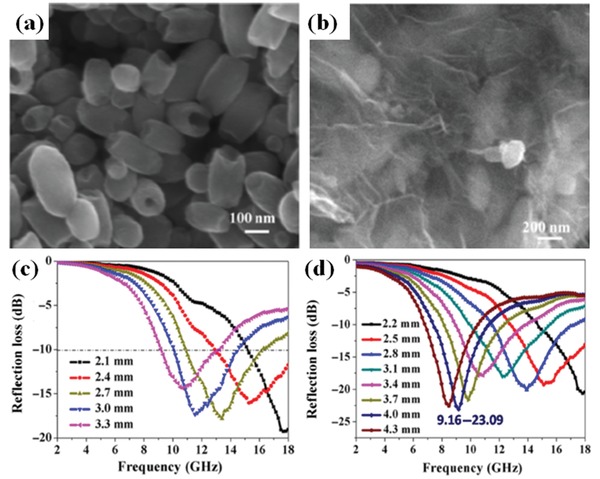
a) SEM images of α‐Fe_2_O_3_ nanorings. b) Morphology of FeNR@rGO rings. Microwave absorption properties of FeNR@rGO with different different compositions and thicknesses: c) GF11, d) GF11‐3h. Reproduced with permission.^102^ Copyright 2016, Springer.

In order to further improve the EM wave absorption performance of the graphene composites, the magnetic metal oxide including MnFe_2_O_4_,^21^ Fe_3_O_4_,^101^, ^102^, ^103^ Co_3_O_4_,^104^ CoO,^105^ NiFe_2_O_4_,^106^ CoFe_2_O_4_, and^107^ ZnFe_2_O_4_ 108 were synthesized by different methods. Zhang et al. reported the preparation of RGO/Fe_3_O_4_/Fe nanoring composites.^102^ The EM wave absorption performance and the microstructures of the prepared composites are shown in **Figure** **16**. The maximum RL of RGO/Fe_3_O_4_/Fe nanoring composites is −23.09 dB and the effective EM wave absorption bandwidth is 3.9 GHz.

**Figure 17:**
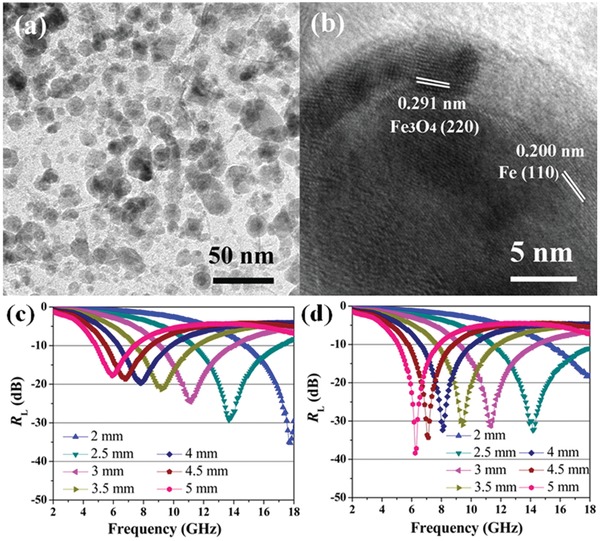
a) Low‐resolution TEM image of the G/Fe_3_O_4_@Fe/ZnO quaternary nanocomposites. b) HRTEM image of an individual Fe_3_O_4_@Fe nanoparticle in the nanocomposites. c) The RL of G/Fe_3_O_4_@Fe ternary nanocomposites. d) The RL of G/Fe_3_O_4_@Fe/ZnO quaternary nanocomposites. Reproduced with permission.^103^ Copyright 2012, American Chemical Society.

Ren et al. synthesized the RGO/Fe_3_O_4_@Fe/ZnO nanocomposites.^103^ The EM wave absorption performances of the synthesized nanocomposites are shown in **Figure** **17**. The nanocomposites have an outstanding effective EM wave absorption bandwidth of 7.3 GHz with the RL below −20 dB. Carbonaceous Co_3_O_4_/Co/RGO composites exhibited the maximum RL of −52.8 dB at 13.12 GHz.^104^ The maximum RL of the CoO/Co/ZnO/graphene composite reached −51.1 dB and the effective EM wave absorption bandwidth was 4.7 GHz.^105^

Ternary NiFe_2_O_4_@SiO_2_@RGO composites had the maximum RL of −42.9 dB and the effective EM wave absorption bandwidth with the RL below −10 dB was 6.2 GHz.^106^ The other ternary CoFe_2_O_4_/RGO/SnS_2_ composites had the maximum RL reached −54.4 dB at 16.5 GHz.^107^ As a common spinel ferrite material, MnFe_2_O_4_ and ZnFe_2_O_4_ have been widely utilized in EM wave absorption fields. ZnFe_2_O_4_@SiO_2_@RGO hierarchical structure composites were prepared by “coating‐coating” method.^108^ The maximum RL of ZnFe_2_O_4_@SiO_2_@RGO composites reached −43.92 dB and the effective EM wave absorption bandwidth was 6.0 GHz. Thus, the prepared ternary graphene composites had excellent EM wave absorption performance in high frequency. The exploitation of ternary graphene composites is an important way for the development of novel and excellent EM wave absorbing materials.

### 3.4 Summary

Graphene and its composites have been considered as promising EM wave absorption materials. However, lots of efforts were still needed to turn the excellent EM wave absorbents into practical applications. Binary heterogeneous graphene composites with conductive metals, ceramics, conductive polymers, and magnetic nanoparticles for EM wave absorption were constructed by many different methods. The integration of graphene and other lossy materials could generally generate synergetic effects, which significantly contributes to the EM wave absorption performance. To further enhance the attenuation of incident EM wave, ternary, and even quaternary composites have been investigated extensively.

## 4 CNTs and Their Composites for EM Wave Absorption

Because of their excellent physical and chemical properties, CNTs have significant potentials to be designed as high‐performance EM wave absorbent. CNTs are perfect electric conductors without magnetism, so their EM wave absorption was mainly originated from the dielectric loss. As a result of the surface effect, CNTs also could be acted as a template to deposit or link with some functional materials, which is very significant for adjusting to their EM wave absorption performance.

### 4.1 CNTs for EM Wave Absorption

The non‐magnetism and poor dispersity of pristine CNTs seriously limit the practical application. Thus, the modifications of pristine CNTs became particularly necessary. Based on the unique structure of rolled graphene wall, defects, and nanotubes, CNTs could be modified by physical or chemical methods.^109^

Sun et al. made a systematic study for the structures and properties of CNTs.^110^ The EM wave RL of 2, 3, and 4 layered aligned CNT films were investigated. With the number of layer increasing, the maximum RL increased from −15.28 to −47.66 dB, and the effective EM wave absorption bandwidth moved to high frequency. Hou et al. synthesized the polymer composites with double‐layer structure. The rare metal doped multi‐walled CNTs and the pure CNTs were designed as the absorbent.^111^ The RL of the polymer composites had two absorption peaks in the range of 2–18 GHz. Meanwhile, the absorption bandwidth of multi‐walled CNTs doped with rare metal was 5.28 GHz.

### 4.2 CNTs/Conductive Polymer Composites for EM Wave Absorption

Conductive polymers, including PANI, polystyrene(PS), PPy and so on, possessed high dielectric loss due to inherent conductivity and charge localization.^112^, ^113^, ^114^, ^115^, ^116^, ^117^, ^118^, ^119^, ^120^ The shielding effectiveness of CNTs/conductive polymer composites mostly were resulted from the EM wave absorption. High conductive PANI/CNTs/PS composites were prepared by in situ polymerization.^112^ The effective EM wave absorption properties with the RL below −10 dB were obtained in the range of 12.4–18.0 GHz. Luo et al. synthesized CNTs/graphene in the matrix of poly‐(dimethylsiloxane). The maximum RL of CNTs/graphene was −55 dB.^113^ The mechanical, thermal and electrical properties of the conductive polymer could be improved by the combination with CNTs. CNTs/conductive polymer composites had significant potentials to design novel and flexible EM wave absorption materials.

### 4.3 CNTs/Ceramics Composites for EM Wave Absorption

**Figure 18:**
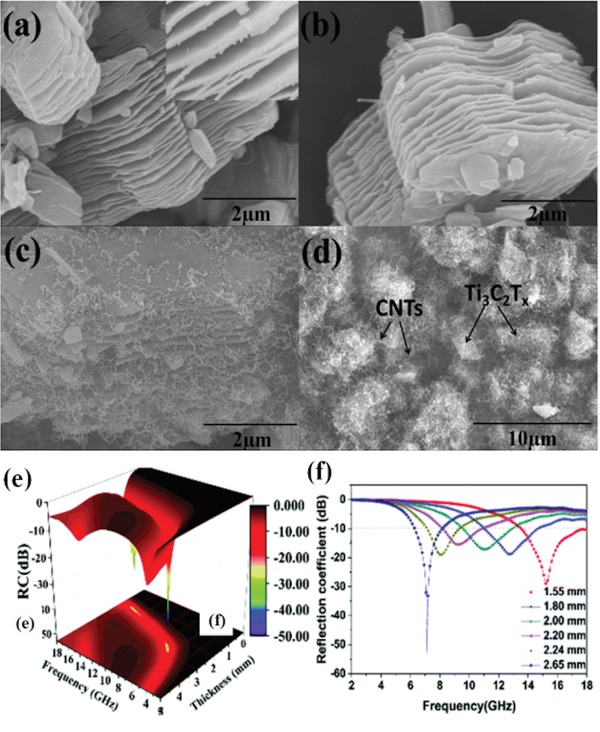
a) SEM images of Ti_3_C_2_T*_x_*. b) SEM images of a‐Ti_3_C_2_T*_x_*. c,d) SEM images of Ti_3_C_2_T*_x_*/CNTs. e) 3D representations and f) theoretical curves of RL versus frequency and thickness of Ti_3_C_2_T*_x_*/CNTs with a filler loading of 35 wt%. Reproduced with permission.^124^ Copyright 2017, The Royal Society of Chemistry.

CNTs/ceramics could effectively coordinate the advantages of CNTs and ceramics. Therefore, CNTs/ceramics composites could be applied in some extremely harsh conditions for high‐frequency EM wave absorption.^121^, ^122^, ^123^, ^124^, ^125^, ^126^, ^127^ Zhang et al. fabricated CNTs/T‐ZnO EM wave absorption materials.^122^ The maximum RL of CNTs/T‐ZnO was −23.00 dB and the effective EM wave absorption bandwidth with the RL below −10 dB was 5 GHz. Ti_3_C_2_T*_x_* MXenes as a new transition metal carbide crystal material showed promising EM wave absorption and shielding effectiveness. Due to the inherent low dielectric loss, Ti_3_C_2_T*_x_* MXenes exhibited weak EM wave absorption performance. Ti_3_C_2_T*_x_* MXenes modified with CNTs were fabricated via a simple catalytic chemical vapor deposition process.^124^ The microstructures and the EM wave absorption performance of the prepared Ti_3_C_2_T*_x_*/CNT nanocomposites are shown in **Figure** **18**. The maximum RL of nanocomposites is −52.9 dB and the effective EM wave absorption bandwidth is 4.46 GHz. The generated multiple interfaces, conductive pathways, and defects of CNTs/ceramics were mainly responsible for the enhanced EM wave absorption performance.^128^, ^129^

### 4.4 CNTs/Magnetic Particles Composites for EM Wave Absorption

It is still worthwhile to indicate that the magnetic particles made more significant contributions to the EM wave absorption. The CNTs were modified by magnetic particles, including Ni,^70^, ^111^ Fe,^131^ Co,^132^ FeCo,^133^ and carbonyl iron^134^ as well as their oxides such as BaFe_12_O_19,_ 135 Fe_3_O_4_,^130^, ^137^ CoFe_2_O_4_,^138^, ^139^ and so on. The larger saturation magnetization and higher Snoek's limits of CNTs/magnetic particles composites guaranteed the better EM wave absorption performance.

**Figure 19:**
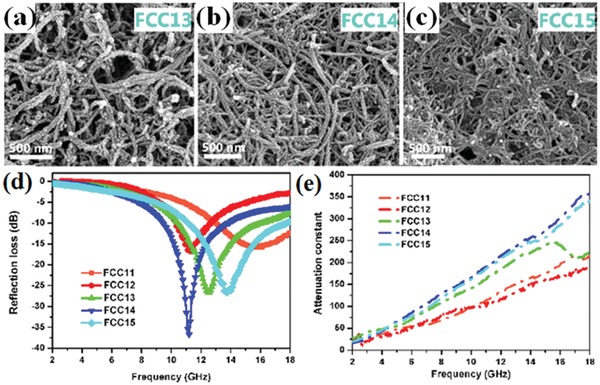
SEM images of pure CNTs and FCCs with various CNT to Fe^3+^mass ratios: a) 1:3 (FCC13), b) 1:4 (FCC14), c) 1:5 (FCC15). d) RL curves of FCC11−FCC15 at 2−18 GHz. e) Plots of attenuation constant versus frequency of FCC11−FCC15 samples. Reproduced with permission.^137^ Copyright 2017, American Chemical Society.

Lin et al. synthesized the CNTs/Fe nanowires composites and the maximum RL of the composites was −22.73 dB.^131^ The CNTs/Co nanoparticles composites were synthesized and the uniform Co nanoparticles were well distributed on the CNTs surface.^132^ The maximum RL of the composites was −36.5 dB. Many magnetic metal alloy composites as like rod‐like FeCo/CNTs were prepared.^133^ The maximum RL of FeCo/CNTs composites was −46.5 dB at 12.56 GHz and the effective EM wave absorption bandwidth was up to 3.92 GHz. The maximum RL of CNTs/CIPs composites was −33.3 dB at 11.4 GHz.^134^ The sandwich microstructure of graphite/BaFe_12_O_19_ shown the maximum RL of −49.5 dB at 11.2 GHz.^135^ Moreover, the bandwidth of RL below −10 dB was 4.2 GHz. The combination of CNTs and W‐type hexagonal ferrite possessed outstanding EM wave absorption performance in 2–8 GHz.^136^ The maximum RL of the CNTs/W‐type hexagonal ferrite composites reached −21.9 dB at 8.5 GHz. The compact‐coated CNTs/Fe_3_O_4_ composites and loose‐coated CNTs/Fe_3_O_4_ composites were prepared by simple solvothermal method.^137^ The microstructures and EM wave absorption performance of the prepared composites are shown in **Figure** **19**. The effective EM wave absorption bandwidth of the prepared composites is 8.3 GHz.

The high density of magnet ferrite restricted its practical applications. Therefore, the magnet ferrite often incorporated with CNTs to decrease the density.^138^, ^139^ Asphalt carbon coated graphene/CoFe_2_O_4_ hollow particles modified CNTs composites were prepared.^138^ The maximum RL of the prepared composites reached −46.8 dB at 11.6 GHz. The effective EM wave absorption bandwidth with the RL below −10 dB was 3.44 GHz. Large‐scale CNTs/CoFe_2_O_4_ nanocomposites were fabricated and their effective EM wave absorption bandwidth was 7 GHz.^139^ The excellent microwave absorption properties mainly resulted from the appropriate impedance matching, strong natural resonance, and multi‐polarizations.

### 4.5 Summary

The excellent RL of the CNT composites mainly resulted from the synergistic effects of conductivity, dielectric, and magnetic loss. First, the incorporation of CNTs and other lossy material can enlarge the absorption bandwidth and strengthen the RL intensity. Secondly, the EM wave absorption performance of the CNTs composites could be enhanced by changing their internal nanostructures. A greater RL value could be obtained from the CNTs composites with a lower additive amount.

## 5 Special Carbon Nanostructures for EM Wave Absorption

The special carbon nanostructures, including carbon nanocapsules,^140^, ^141^ carbon nanospheres,^142^, ^143^, ^144^, ^145^, ^146^, ^150^ mesoporous carbons,^147^, ^148^, ^149^ MOF‐derived porous carbons,^152^, ^153^, ^154^, ^155^, ^156^ carbon nanowires or nanorods,^157^, ^158^, ^159^, ^160^ and onion‐like or bowl‐like nano carbons,^161^, ^162^ have been investigated in fields of EM wave absorption.

### 5.1 Core‐Shell Carbon Nanocapsules for EM Wave Absorption

Compared to the pure metallic magnets, core‐shell carbon nanocapsules with magnetic particles have lower density and larger resistivity. Core‐shell carbon nanocapsules could be applied in high‐frequency EM wave absorption. The (Fe, Ni)/C nanocapsules composites were fabricated and their maximum RL was −26.9 dB at 16 GHz.^140^ The EM wave absorption bandwidth with the RL below −20 dB could be achieved in the range of 13.6–16.6 GHz. The maximum RL for carbon‐coated Fe[Fe(C)] nanocapsules was −43.5 dB at 9.6 GHz.^141^ The excellent EM wave absorption properties mainly resulted from the appropriate impedance matching, strong natural resonance, as well as multi‐polarization. The carbon shell made the metal nanoparticles free from oxidation and suppresses the eddy current loss.

### 5.2 Core‐Shell Carbon Nanosphere for EM Wave Absorption

**Figure 20:**
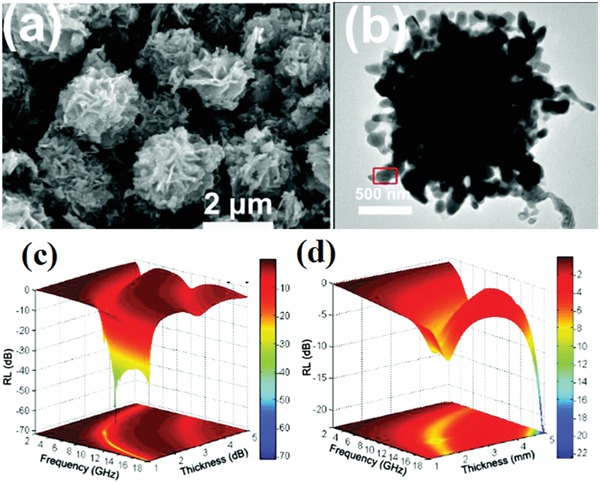
a,b) SEM and TEM images of rose‐like Fe@C hierarchical structure. c,d) RL 3D diagram of rose‐like Fe@C and pure iron in different thickness. Reproduced with permission.^146^ Copyright 2018, The Royal Society of Chemistry.

The EM wave absorption performance of the carbon‐encapsulated cobalt nanoparticles [Co(C)] could be improved by modulating the impedance matching between carbon material and free space. The inner crystal core was completely encapsulated by an outer carbon shell.^142^, ^150^ Core/shell structured carbon/ZnO composites were prepared by a hydrothermal method.^143^ The EM wave absorption ability was enhanced by the heterogeneous interface between carbon spheres and ZnO nanoparticles. The maximum RL of the carbon/ZnO composites reached −52 dB. Porous Ni/C composites were synthesized and their maximum RL of Ni/C composites was −51.8 dB.^136^ Hierarchical rose‐like Fe@C composites with porous architecture were fabricated. The microstructures and EM wave absorption performance of the fabricated composites are shown in **Figure** **20**.^146^ The maximum RL of the fabricated composites is −71.47 dB, which is higher than RL values of other composites. The EM wave absorption mechanisms of the core/shell carbon nanostructure originated from orientational polarization, space charge polarization, and electrical conductivity.^142^, ^143^, ^146^

### 5.3 Mesoporous Carbon Nanostructures for EM Wave Absorption

Recently, the ordered mesoporous carbon (OMC) have been widely designed as EM wave absorption materials. Double‐layer OMC/paraffin composites were fabricated by hydrothermal reactions and pyrolysis.^147^ The maximum RL of double‐layer OMC/paraffin composites was −28.5 dB. 1D α‐Fe_2_O_3_/OMC hybrids were fabricated by facial hydrothermal reaction.^148^ The unique heterogeneous structures of α‐Fe_2_O_3_/OMC hybrids significantly improved the dielectric loss. The effective EM wave absorption bandwidth could be obtained in the range of 10.5–16.5 GHz. Besides, the maximum RL value of OMC/Ni_2_O_3_ composites was −39 dB at 10.9 GHz.^149^ The EM wave could be effectively attenuated by the mesoporous carbon structure.

### 5.4 MOF‐Derived Carbon Nanoporous for EM Wave Absorption

**Figure 21:**
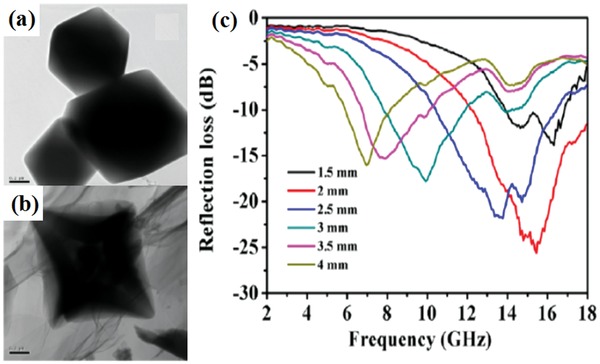
a) TEM images of MOF‐53(Fe). b) TEM images of MOF‐53(Fe)/RGO composite. c) The RL curves of MOF‐53(Fe)/RGO composite. Reproduced with permission.^152^ Copyright 2016, Elsevier.

MOF‐derived carbonaceous metal oxides have unique nanoporous structures and superior properties.^151^ MOF‐53(Fe)/RGO composites were synthesized successfully.^152^ The structure and EM wave absorption performance of MOF‐53(Fe)/RGO composites are shown in **Figure** **21**. The maximum RL of the composites reaches −25.8 dB at 15.4 GHz and the effective EM wave absorption bandwidth is 5.9 GHz. Moreover, the maximum RL of MOF‐derived nanoporous porous Co/C composites was −35.3 dB and the effective EM wave absorption bandwidth was 5.80 GHz.^153^ Core‐shell Co/nanoporous carbon(NPC)/TiO_2_ was fabricated and the maximum RL of −51.7 dB achieved.^154^ Porous CuO/carbon composites were synthesized by nitrate impregnation into a MOF template.^155^ The maximum RL of porous CuO/carbon composites was −57.5 dB and the RL below −10 dB could be gained in the range of 13–17.7 GHz. By annealing titanium‐based MOFs(MIL‐125), the novel carbon nanoporous material (TiO_2_/C) was prepared.^156^ The maximum RL of −49.6 dB and the effective EM wave absorption bandwidth of 4.6 GHz could be obtained. The outstanding EM wave absorption performance of the MOF‐derived carbon nanoporous mainly originated from the dielectric loss. With the incorporation of some dielectric or magnetic loss materials, the impedance matching of the MOF‐derived carbon nanoporous could be improved greatly.

### 5.5 Core‐Shell Nanorods/Nanowires for EM Wave Absorption

SiC@C core‐shell nanowires possessed high‐performance EM wave absorption ability. The thickness of carbon shell could be modulated from 4 to 20 nm.^157^ The maximum RL of SiC@C core‐shell nanowires was −50 dB at 12 GHz and the effective absorption bandwidth was 8 GHz. Porous core/shell Fe_3_O_4_/carbon nanorods composites were fabricated and the Fe_3_O_4_ core exhibited a characteristic of porosity.^158^ The thickness of the carbon shell was decreased to about 2.5 nm and its degree of graphitization was enhanced. The maximum RL of composites was −27.9 dB at 14.96 GHz. The maximum RL of carbon nanofibers‐Fe composites was −67.5 dB at 16.6 GHz.^159^ The twin carbon nanocoils (T‐CNCs) composites were synthesized by the decomposition of acetylene over nickel nanoparticles.^160^ The magnetic parameters of the composites demonstrated that the good absorption properties of T‐CNCs attributed to the dielectric loss rather than the magnetic loss. Moreover, the maximum RL and the effective EM wave bandwidth were −36.09 dB and −20 GHz, respectively. The combination of core‐shell carbon nanowires/nanorods and lossy materials generated dipole polarization and interfacial polarization, which were responsible for the enhanced EM wave absorption.

### 5.6 Other Carbon Nanostructures for EM Wave Absorption

**Figure 22:**
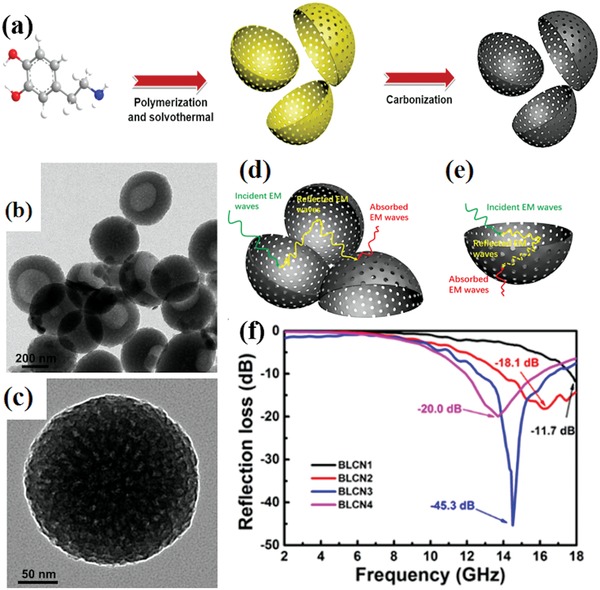
a) Schematic illustration for the synthesis process of BLCNs. b) TEM images of BLCNs. c) Magnified TEM image for an individual BLCNs. d) Mechanism of EM microwave absorption in BLCNs: consumption via multiplied reflections among BLCNs. e) consumption in pores or voids. f) RL for BLCN3 with thickness of 1.5 mm. Reproduced with permission.^162^ Copyright 2017, American Chemical Society.

Onion‐like carbon (OLC) had unique structure and EM wave absorption performance.^161^ The effective EM wave absorption bandwidth could be obtained in a wide frequency range (11.9–16.3 GHz). The special bowl‐like carbon nanoparticles (BLCNs) and their EM wave absorption performance are shown in **Figure** **22**.^162^ The maximum RL and the effective bandwidth of the BLCNs are −45.3 dB and 4.2 GHz, respectively. The incident EM wave could be absorbed by the interfacial and dipole polarization of the special carbon nanostructures.

### 5.7 Summary

The special carbon nanostructure and their composites have a nanocarbon thin film at the surface of lossy materials. The nanocarbon films generate dual dielectric relaxation including the Debye dipolar and interfacial polarization on the interfaces. In addition, the special carbon nanostructures combined with dielectric or magnetic loss materials are benefit to the improvement of impedance matching. So, the incident EM wave could be effectively attenuated in the special carbon nanostructures.

## 6 Conclusion and Prospects

Based on the above summaries, the recent advanced developments of carbon nanostructures for EM wave absorption were comprehensively introduced and summarized. Due to the outstandingly intrinsic properties including low density, the great specific surface area, excellent electrical conductivity as well as dielectric properties, carbon nanostructures have a great potential in the field of EM wave absorption. The synergistic effect of the carbon nanostructures could be modulated by incorporating other magnetic or dielectric components. The prepared multicomponent carbon nanostructures composites have made significant progress in EM wave absorption.

However, there are many challenges of multicomponent carbon nanostructures composites for EM wave absorption: (1) the combination of structure and function design technologies, (2) the lightweight design with excellent absorption performance, (3) the broadband and strong EM wave absorption in low frequency, (4) the excellent environmental stability of carbon nanostructures under harsh conditions, 5) clear EM wave absorption mechanisms for carbon nanostructures. The challenges are needed to be solved urgently. Hence, the design and preparation of novel multicomponent carbon nanostructures with high‐performance EM wave absorption ability should be regarded as the orientation of studies and applications. Moreover, the already existing EM wave absorption materials could effectively absorb the centimeter wave in 8–18 GHz. With the development of metric wave and millimeter‐wave military radar technology, the EM wave absorption materials should be compatible with the metric wave, centimeter wave, even infrared and laser. Therefore, the design of carbon nanostructures for multi‐band EM wave absorption would become an important research trend.

In conclusion, carbon nanostructures and its composites show excellent EM wave absorption properties in high frequency. However, there are still many problems to be solved in order to realize its practical application. We believe that these challenges and problems will be solved continuously with the joint efforts of scientists around the world. The carbon nanostructures as novel EM absorbing materials have the great potential to change the world and represent the brilliant future of humanity.

## Conflict of Interest

The authors declare no conflict of interest.
